# Central role of the p53 pathway in the noncoding-RNA response to oxidative stress

**DOI:** 10.18632/aging.101341

**Published:** 2017-12-12

**Authors:** Paola Fuschi, Matteo Carrara, Christine Voellenkle, Jose Manuel Garcia-Manteiga, Paolo Righini, Biagina Maimone, Elena Sangalli, Francesco Villa, Claudia Specchia, Mario Picozza, Giovanni Nano, Carlo Gaetano, Gaia Spinetti, Annibale A. Puca, Alessandra Magenta, Fabio Martelli

**Affiliations:** ^1^ Molecular Cardiology Laboratory, IRCCS Policlinico S. Donato, 20097, San Donato Milanese, Milan, Italy; ^2^ Center for Translational Genomics and BioInformatics, IRCCS San Raffaele Scientific Institute, 20132, Milan, Italy; ^3^ Operative Unit of Vascular Surgery I, IRCCS Policlinico S. Donato, 20097, San Donato Milanese, Milan, Italy; ^4^ Cardiovascular Research Unit, IRCCS MultiMedica, 20138, Milan, Italy; ^5^ Department of Molecular and Traslational Medicine, University of Brescia, Brescia, 25123, Italy; ^6^ Vascular Pathology Laboratory, Istituto Dermopatico dell'Immacolata-IRCCS, FLMM, Rome, Italy; ^7^ University of Milan, 20133, Milan, Italy; ^8^ Division of Cardiovascular Epigenetics, Department of Cardiology, Goethe University, Frankfurt am Main, 60596, Germany; ^9^ Department of Medicine and Surgery, University of Salerno, 84084, Salerno, Italy

**Keywords:** oxidative stress, long noncoding RNAs, microRNAs, p53, endothelium

## Abstract

Oxidative stress plays a fundamental role in many conditions. Specifically, redox imbalance inhibits endothelial cell (EC) growth, inducing cell death and senescence. We used global transcriptome profiling to investigate the involvement of noncoding-RNAs in these phenotypes. By RNA-sequencing, transcriptome changes were analyzed in human ECs exposed to H_2_O_2_, highlighting a pivotal role of p53-signaling. Bioinformatic analysis and validation in p53-silenced ECs, identified several p53-targets among both mRNAs and long noncoding-RNAs (lncRNAs), including MALAT1 and NEAT1. Among microRNAs (miRNAs), miR-192-5p was the most induced by H_2_O_2_ treatment, in a p53-dependent manner. Down-modulated mRNA-targets of miR-192-5p were involved in cell cycle, DNA repair and stress response. Accordingly, miR-192-5p overexpression significantly decreased EC proliferation, inducing cell death. A central role of the p53-pathway was also confirmed by the analysis of differential exon usage: Upon H_2_O_2_ treatment, the expression of p53-dependent 5′-isoforms of MDM2 and PVT1 increased selectively. The transcriptomic alterations identified in H_2_O_2_-treated ECs were also observed in other physiological and pathological conditions where redox control plays a fundamental role, such as ECs undergoing replicative senescence, skeletal muscles of critical limb-ischemia patients and the peripheral-blood mononuclear cells of long-living individuals. Collectively, these findings indicate a prominent role of noncoding-RNAs in oxidative stress response.

## INTRODUCTION

Redox homeostasis plays a fundamental role in endothelial cell (EC) function and its imbalance elicits oxidative stress that has a causative role in many vascular diseases [1, 2]. Augmented EC oxidative stress is a result of increased production of reactive oxygen species from intracellular enzymes such as NADPH oxidase and uncoupled eNOS, as well as from mitochondrial respiration, in the absence of counter-acting antioxidant defenses. As redox imbalance poses a major threat to EC function and survival, it triggers specific transcriptional and post-transcriptional modulation of gene expression [3, 4].

Aberrant control of gene expression underpins most pathologies. Thus, it is of pivotal importance to investigate how gene expression is regulated by the underlying factors of diseases, starting from oxidative stress.

Several hubs of cell response to oxidative stress have been identified, such as NRF2 and FOXO1, that increase the antioxidant defenses, and NFkB, that induces the transcription of pro-inflammatory cytokines [3, 4]. Another pivotal player is p53: it is activated by a multitude of stress stimuli, including reactive oxygen species, and, in turn, orchestrates an extremely complex anti-proliferative and pro-apoptotic transcriptional program [5].

Investigating the responses triggered by cell exposure to a single oxidative stress stimulus, such as a H_2_O_2_ bolus, might be instrumental to the identification of the molecular mechanisms underpinning more complex situations characterized by redox imbalance, such as proliferative senescence [6-8], limb ischemia [9-11] and aging [12, 13].

While the role of protein-coding transcripts in cell response to oxidative stress has been extensively studied [3, 4, 14], the importance of non-protein-coding RNAs (ncRNAs) has started to emerge only recently [9, 15].

ncRNAs are extremely heterogeneous [16]. Some RNAs, mostly with housekeeping functions, have been long known, such as ribosomal RNAs, transfer RNAs, as well as small nuclear and nucleolar RNAs. However, increasing attention has been dedicated in the last few years to two classes of regulatory ncRNAs: microRNAs (miRNAs) and long ncRNAs (lncRNAs).

miRNA are single-stranded, about 22 nt RNAs that regulate gene expression mostly by forming partial hybrids with target mRNAs and, in this manner, lowering their stability and/or translation efficiency [17]. Profiling of miRNA expression in H_2_O_2_-treated EC identified miR-200c as upregulated by oxidative stress. miR-200c induces EC apoptosis and senescence *via* ZEB1 inhibition and by disrupting the regulatory loop among SIRT1, FOXO1, and eNOS [18, 19].

LncRNAs are >200 nt long RNAs that are very heterogeneous both for their genomic organization and for their molecular mechanism of action [20]. They can be natural antisense (AS) expressed from the opposite strand of mRNAs, generated by intergenic regions (lincRNAs), localized in introns of annotated genes and transcribed from enhancers regulatory elements (eRNAs). Mechanistically, they can regulate gene expression epigenetically or acting as scaffolds for transcriptional repressors and activators. Certain lncRNAs can play a role in nuclear body compartmentalization. Other lncRNAs regulate gene expression post-transcriptionally, modulating mRNA splicing and stability with different mechanisms. While lncRNAs are often expressed at low levels, some of them are sufficiently abundant to act as decoys for microRNAs (competing endogenous RNAs) and possibly other regulatory molecules.

LncRNAs regulate a variety of cell functions that might impact upon cell response to oxidative stress [15, 20]. In this study, we attempted to identify the involvement of lncRNAs and miRNAs in the molecular mechanisms underpinning EC response to oxidative stress. We profiled the transcriptomic changes induced by H_2_O_2_ treatment in EC, and validated the identified players and pathways with molecular and functional experiments. We also extended our investigation to other physio-logical and pathological conditions where redox imbalance plays a prominent role, suggesting that the ncRNAs identified might play a broader role in oxidative stress response.

## RESULTS

### H_2_O_2_ treatment inhibits endothelial cell growth

To analyze the transcriptomic changes induced by EC exposure to oxidative stress, human umbilical cord EC (HUVEC) were cultured in the presence or absence of 200 μM H_2_O_2_ for 16 hrs or 36 hrs, and total RNA was extracted. Cells derived from three different batches of HUVEC were used, in order to minimize the identification of transcriptomic responses characteristic only of specific EC isolates. To control the efficacy of the H_2_O_2_ treatment, HUVEC plates grown in parallel were assayed. Cell cycle profile analysis and bromo-deoxyuridine (BrdU) incorporation assay by FACS showed that, in the adopted condition, H_2_O_2_ treatment strongly decreased EC proliferation (Fig. S1A and B). The percentage of sub-G1 dead cells remained below 3% following H_2_O_2_ treatment, indicating a mostly cytostatic response (Fig. S1A and C). HUVEC treated with solvent alone for 16 hrs were chosen as control to avoid the possibly confounding effects of cell confluency observed at 36 hrs. As further control, the expression of known H_2_O_2_ responsive genes was tested in the isolated RNAs by qPCR. As expected [9, 15], CDKN1A and miR-200c-3p expression were increased, while ZEB1 levels were reduced (Fig. S2).

To analyze global changes in the transcriptome, mRNAs, lncRNAs and miRNAs profiles were measured by RNA-sequencing.

### mRNA changes induced by H_2_O_2_ treatment indicate p53-pathway relevance

A total of 9 barcoded cDNA libraries were prepared from rRNA-depleted RNA derived from HUVEC cells exposed to H_2_O_2_ for 16 and 36 hrs or to solvent alone for 16 hrs. Reads were generated by next-generation sequencing yielding ≈ 1 billion forward and reverse reads after filters for quality and unique alignment to the human hg19 reference genome were applied.

Gene level analysis provided a total of ≈ 15 000 expressed genes and 76% of them were annotated as protein coding. Of these, ≈ 2 300 were significantly differentially expressed at 16 hrs (Dataset S1) and ≈ 3 100 at 36 hrs (Dataset S2); about 1800 genes were in common between the two timepoints (Dataset S3). These data indicate a profound effect of oxidative stress on HUVEC gene expression.

As expected, genes previously described as modulated by oxidative stress, such as CDKN1A, HMOX1, MCM6, GADD45A, and FAS [15] were among the most significantly modulated. This analysis was further validated measuring a subset of mRNAs by qPCR in independent samples of H_2_O_2_ treated HUVEC (Fig. S3).

**Figure 1:**
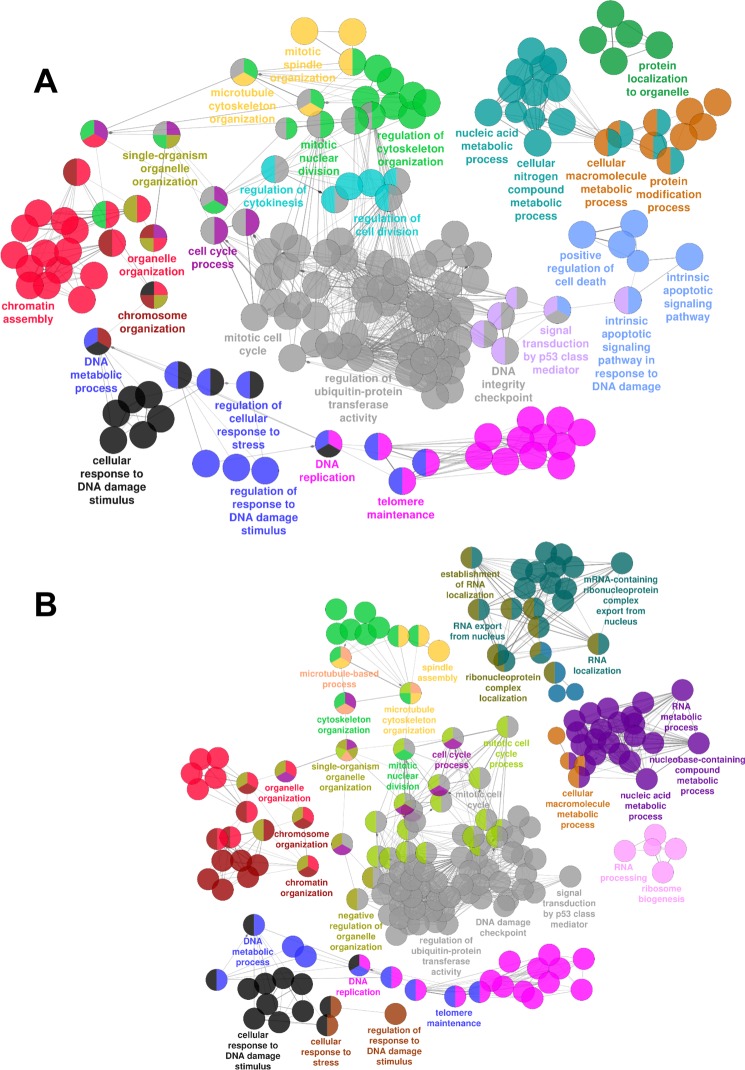
mRNAs differentially expressed upon HUVEC exposure to H_2_O_2_ Gene Ontology enrichment analysis of the transcriptomic changes induced by HUVEC treatment with H_2_O_2_ as assessed by rRNA-depleted RNA-sequencing. Circles represent specific ontology terms or KEGG pathways that were significantly enriched in the list of differentially expressed genes after 16 hrs (**A**) or 36 hrs (**B**) of H_2_O_2_ treatment (n= 3). Edges represent term connections within the ontology tree and colors highlight terms correlated in meaning. Terms are captioned if they are the most significant of the group or if they show a biological meaning connected to the system under analysis.

In order to identify relevant biological functions, enriched biological processes and pathways were identified by ClueGO [21]. Gene ontology enrichment analysis at both 16 hrs (Fig. 1A) and 36 hrs (Fig. 1B) shows an enrichment in terms related to cell cycle, mitotic spindle/microtubules, DNA damage/stress response, apoptosis, chromatin and telomere integrity, RNA and nucleic acid metabolism, as well as p53 signaling.

**Figure 2:**
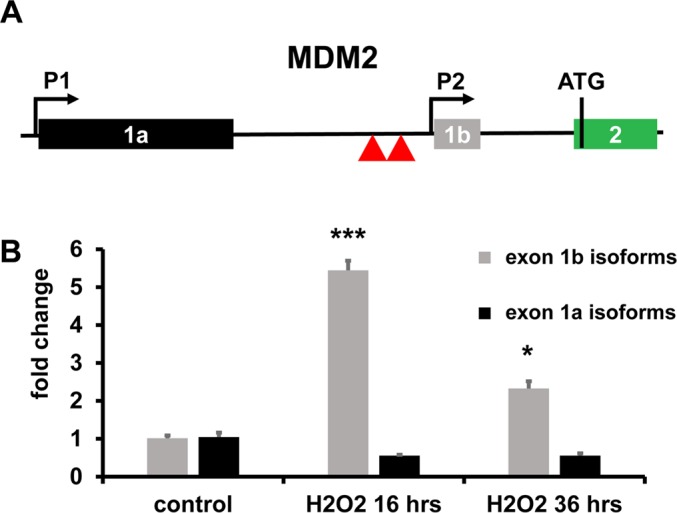
Differential MDM2 exon usage (**A**) Genomic structure of the human MDM2 promoter region. The locus has two independent promoters before exons 1a (P1) and 1b (P2), yielding long- and short-5′ UTR isoforms, respectively. Translation start site (ATG) in exon 2 is indicated. Red triangles indicate p53 protein binding sites involved in P2 activation. (**B**) In H_2_O_2_ treated HUVEC, short-5′ UTR isoforms of MDM2 were induced, as assessed by qPCR using primers spanning from exon 1b to exon 2. Modulation of long-5′ UTR isoforms of MDM2 was not statistically significant, as assessed by qPCR using primers spanning from exon 1a to exon 2. The bar graph represents average values ±SEM (n= 3; ^*^p<0.05, ^***^p<0.001).

Among the top modulated genes, both at 16 hrs and at 36 hrs, it was identified MDM2, a crucial negative regulator of p53, as well as a p53-induced gene [22]. A variety of MDM2 transcripts have been described [23]. Specifically, the expression of MDM2 transcripts with short 5′UTRs, including exon 1b, has been shown to be largely p53-dependent, unlike that of MDM2 isoforms with long 5′UTR, using exon 1a [23] (Fig. 2A). Of note, short 5′UTRs isoforms are more efficiently translated, establishing a p53/MDM2 negative feed-back loop [23, 24]. Differential exon usage analysis (Fig. S4) and qPCR validation (Fig. 2B), showed that, upon H_2_O_2_ treatment, MDM2 transcripts including exon 1b accumulated significantly, while transcripts including exon 1a did not (Fig. 2B).

### LncRNA modulations induced by oxidative stress

**Figure 3:**
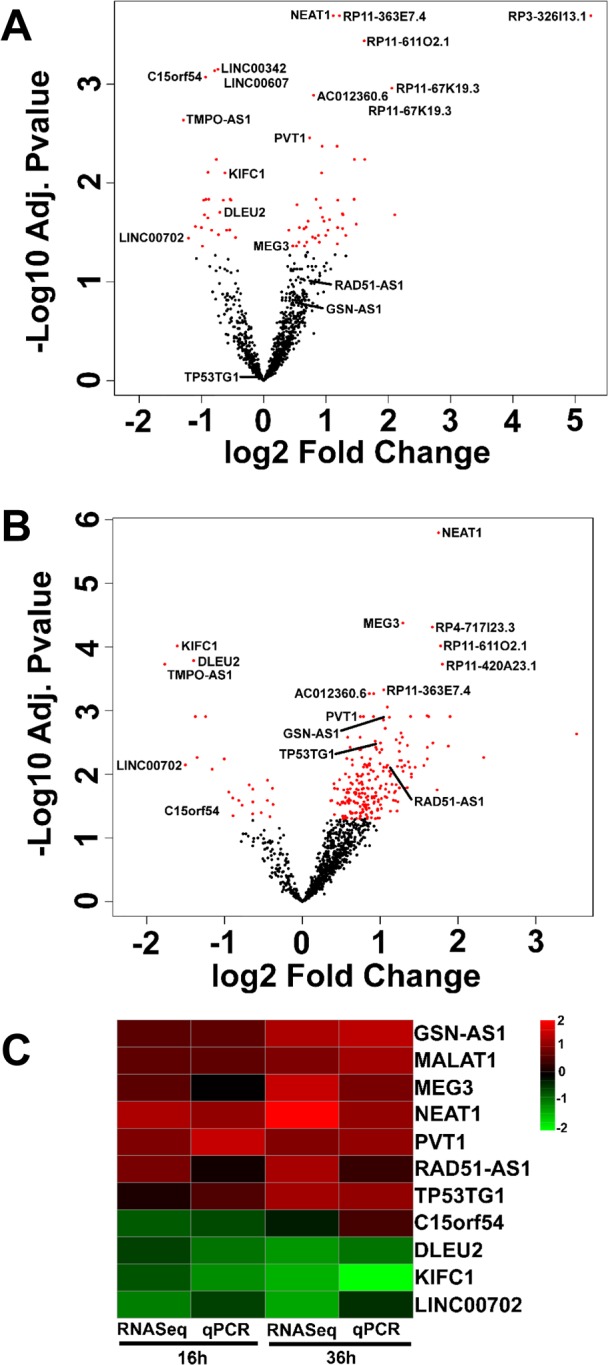
LncRNAs differentially expressed upon HUVEC exposure to H_2_O_2_ HUVEC were exposed to H_2_O_2_ for 16 hrs (**A**) and 36 hrs (**B**) and lncRNA expression was measured by rRNA-depleted RNA-sequencing (n= 3). Volcano plots show adjusted p values in a negative Log_10_ scale and fold changes in a log_2_ scale. LncRNAs significantly (adjusted p values <0.05) modulated by H_2_O_2_ treatment are indicated in red. The names of top 10 modulated lncRNAs and of qPCR validated lncRNAs are indicated. (**C**) In independent HUVEC cultures treated as in **A** and **B** (n= 3), the modulation of the indicated lncRNAs was assayed by qPCR. The heat map shows modulated lncRNAs as log_2_ values. Green= down-modulation; red= up-regulation.

Gene expression analysis identified ≈ 1 000 lncRNAs, most lacking basic functional annotation. Of these, 101 and 208 were differentially expressed significantly at 16 hrs (Fig. 3A and Dataset S1) and at 36 hrs (Fig. 3B and Dataset S2), respectively, with 72 lncRNAs in common between the two timepoints (Dataset S4). Thus, data indicated a strong effect of oxidative stress on the expression also of ncRNAs in HUVEC. This analysis was validated measuring a subset of lncRNAs by qPCR in independent samples of H_2_O_2_-treated HUVEC. The heatmap in Fig. 3C shows side by side rRNA-depleted RNA-sequencing and qPCR data expressed in a log_2_ scale. Data expressed in a linear scale are shown in Fig. S5.

PVT1 is a potential oncogene that has been implicated in a variety of cancers, participating in DNA rearrangements, interacting with MYC and encoding miRNAs [25]. PVT1 undergoes extensive alternative splicing to produce a wide variety of mature transcripts. In particular, a difference in exon 1a/1b usage has been observed after treatment with p53-inducing agents in cancer cells [26]. Likewise, in HUVEC, H_2_O_2_ treatment led to a significant increase of p53-dependent exon 1b, while exon1a induction did not reach statistical significance (Fig. S6).

### miRNA/target interactions identify miR-192-5p as a hub of endothelial cell response to H_2_O_2_

To analyze miRNA expression changes induced by EC exposure to oxidative stress, RNAs derived from HUVEC treated with H_2_O_2_ for 16 hrs were analyzed by small RNA-sequencing.

**Figure 4:**
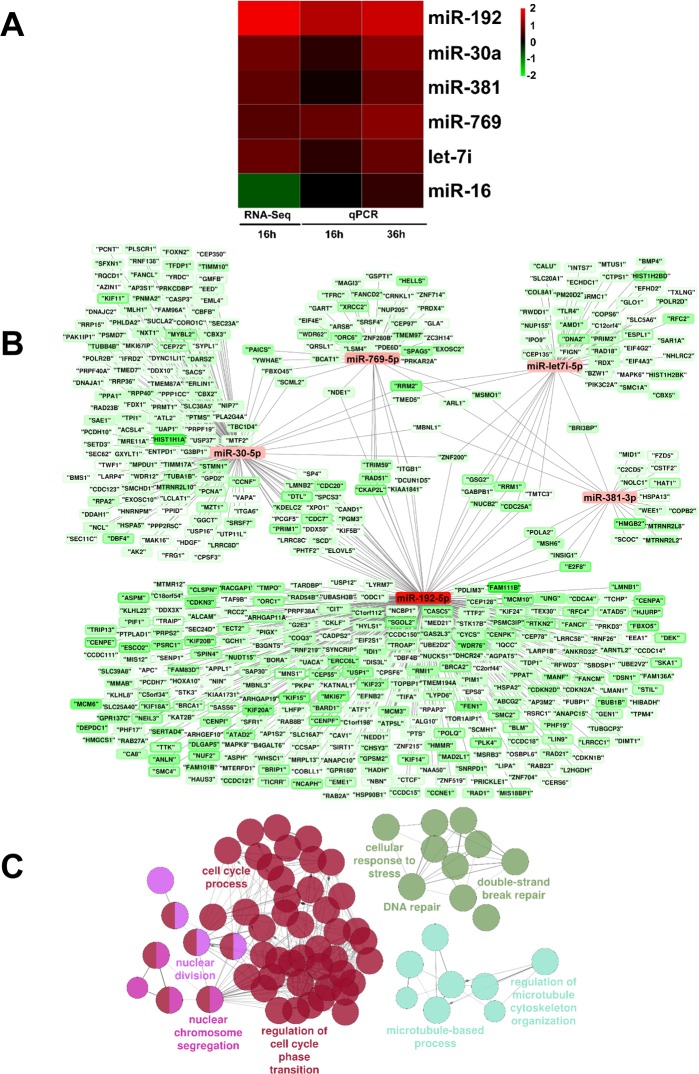
miRNAs differentially expressed upon HUVEC exposure to H_2_O_2_ (**A**) HUVEC were exposed to H_2_O_2_ for 16 hrs and miRNA expression was measured by small RNA-sequencing (n= 3). Validation was performed by qPCR in independent HUVEC cultures treated with H_2_O_2_ for 16 hrs and 36 hrs (n= 3). The heat map shows modulated lncRNAs as log_2_ values. Green= down-modulation; red= up-regulation. (**B**) Interactions between qPCR-validated miRNAs and their potential targets, as reported by miRTarBase, that showed a significant down-regulation at both 16 hrs and 36 hrs time points in rRNA-depleted RNA-sequencing data. Results were represented using Cytoscape: For each gene, the inner color represents the log_2_ fold change at 16 hrs and the border color represents the log_2_ fold change at 36h. (**C**) miR-192-5p targets enrichment analysis. Enrichment analysis performed with ClueGO on miR-192-5p targets that showed a significant down regulation at both time points in rRNA-depleted RNA-sequencing data. Circles represent specific ontology terms or KEGG pathways that are significantly enriched. Edges represent terms connections within the ontology tree and colors highlight terms correlated in meaning. Terms are captioned if they are the most significant of the group or if they show a biological meaning connected to the system under analysis.

After trimming filters for quality and unique alignment to the human hg19 reference genome were applied, ≈ 7.5 million reads were obtained allowing the detection of ≈ 100 miRNAs (DataSet S5). Among miRNAs that escaped detection there were miR-200-family members. Differential expression analysis identified 9 significantly modulated miRNAs (DataSet S5). Quite surprisingly, miR-16 appeared to be modulated by H_2_O_2_, in disagreement with previous findings [9, 18]. qPCR validation on RNAs derived from independent experiments showed that indeed miR-16 was not modulated by H_2_O_2_. Conversely, miR-192-5p, miR-30a-5p, miR-381-3p, miR-769-5p and let7i-5p were significantly up-regulated, both at 16 and 36 hrs of H_2_O_2_ treatment (Fig. 4A and supplementary Fig. S7 in linear scale). Bioinformatic analysis using miRTarBase [27] and Cytoscape [28] highlighted interactions between validated miRNAs and reciprocally modulated genes that had been identified as targets of these miRNAs. Fig. 4B identified miR-192-5p as a hub of miRNA/target interactions in EC response to H_2_O_2_. Accordingly, gene ontology analysis of potential miR-192-5p targets displayed an enrichment in terms related to cell cycle, DNA damage/stress response and microtubules (Fig. 4C).

Next, we investigated whether miR-192-5p induction could be prevented by reactive oxygen species scavengers. To this aim, we treated HUVEC with the alkylating agent 1,3-bis(2 chloroethyl)-1-nitrosourea (BCNU), an inhibitor of glutathione reductase that blocks the conversion of oxidized to reduced glutathione [29], in the presence or absence of N-acetyl-L-cysteine (NAC) to prevent the decrease in intracellular reduced glutathione induced by BCNU [29]. Incubation of HUVEC with 0.25 mM BCNU for 2 h increased miR-192-5p levels and this phenomenon was inhibited by preincubation with 10 mM NAC (Fig. S8).

**Figure 5:**
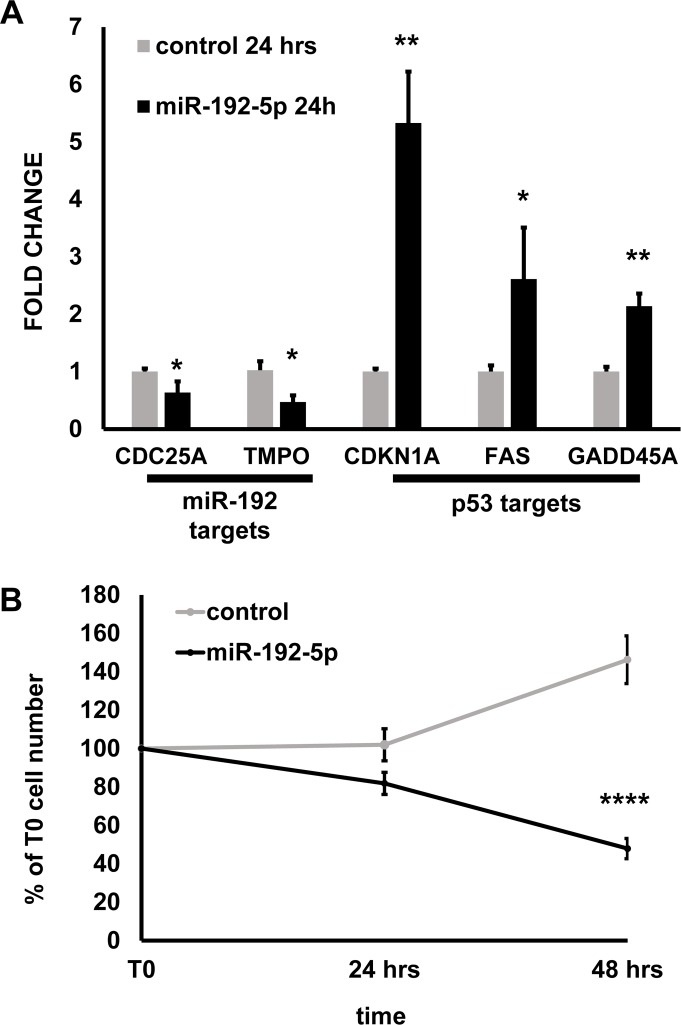
miR-192-5p overexpression inhibits EC growth HUVEC were transfected with miR-192-5p mimic or control oligonucleotides. The next day, cells were plated at 200 000 cells/p3 plate density (T0) and harvested 24 and 48 hrs later. (**A**) The expression of the indicated genes was measured by qPCR at 24 hrs from T0. The bar graph represents average values ±SEM (n= 3; ^*^p<0.05 ^**^p<0.01). (**B**) Cells were counted at 24 hrs and 48 hrs from plating (T0) and plotted as % of the T0 cell number. miR192-5p expression inhibited HUVEC growth significantly. The graph represents average values ±SEM (n=8 independent transfections; ^****^ p<0.0001).

To further validate the relevance of miR-192-5p role in EC growth, we tested the effect of miR-192-5p overexpression in the absence of oxidative stress. miR-192-5p expression decreased the mRNA levels of two predicted targets, TMPO and CDC25A (Fig. 5A), that were also down-modulated in H_2_O_2_ treated cells (Dataset S3). miR-192-5p expression has been shown to activate p53 pathway in cancer cells [30-33]. Likewise, its expression in HUVEC induced the expression of the p53 targets CDKN1A, FAS and GADD45A (Fig. 5A), that were also increased in H_2_O_2_ treated cells (Dataset S3). In keeping with these findings, miR-192-5p dramatically decreased HUVEC growth, inducing cell death (Fig. 5B).

### p53 implication in the regulation of noncoding RNAs by H_2_O_2_

Given the importance of p53 for mRNA modulation by oxidative stress highlighted by the gene ontology analysis (Fig. 1), we investigated whether the same was true for lncRNAs.

In order to identify p53 binding sites in the regulatory regions of the identified H_2_O_2_-modulated lncRNAs, we took advantage of a publicly available ChIP-sequencing (ChIP-seq) datasets for p53.

Since no p53 ChIP-seq datasets have been published so far in ECs, we used data derived from osteosarcoma cells treated with nutilin, an activator of p53 [34]. The region encompassing 5 kbp upstream and 2 kbp downstream the transcription start site of each lncRNA was analyzed for the presence of significant (False Discovery Rate, FDR,< 0.001) peaks corresponding to a consensus p53 binding-site. As expected, known p53 targets such as CDKN1A and GADD45A displayed very significant peaks (Fig. S9A and B). It was found that 29 lncRNAs modulated by H_2_O_2_ in HUVEC also displayed at least one significant peak for p53 protein in nutilin treated osteosarcoma cells (Supplementary Dataset S6 and Fig. S9C, D and E). Patterns of alternative exon usage for PVT1 and MDM2 were observed as well (Fig. S9E and F). This analysis strongly suggests that p53 might regulate the identified lncRNAs in H_2_O_2_ treated ECs.

**Figure 6:**
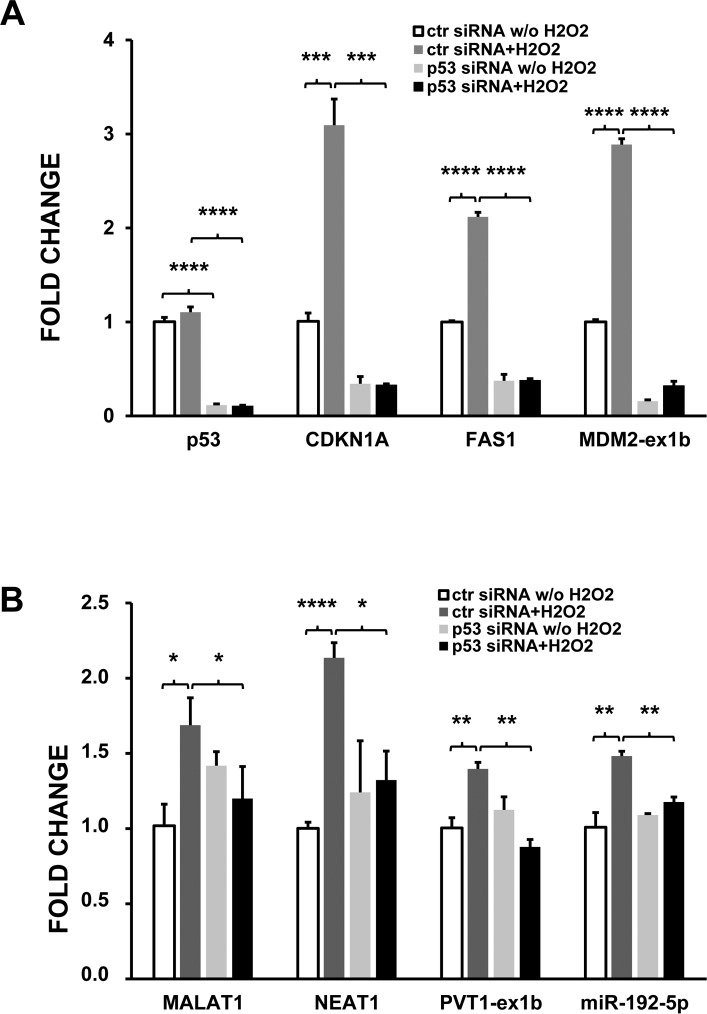
p53 silencing inhibits the induction of target ncRNAs by H_2_O_2_ HUVEC were transfected with p53 or control (ctr) siRNAs and, 24 hrs later, treated with either 200 μM H_2_O_2_ or solvent alone. After 16 hrs, total RNA was extracted and the indicated RNAs analyzed by qPCR. (**A**) coding RNAs; (**B**) ncRNAs. The bar graphs show average fold changes ±SEM of the indicated RNAs referred to untreated cells transfected with control siRNA (n= 3; ^*^p<0.05, ^**^p<0.01, ^***^p<0.001).

To corroborate this hypothesis, modulation by oxidative stress of a subset of target genes was analyzed by qPCR in HUVEC where p53 had been knocked down. HUVEC transfection with p53 siRNAs significantly decreased the corresponding transcript (Fig. 6A). As expected, the mRNA levels of known p53 targets, such as CDKN1A and FAS1, were significantly decreased by p53 silencing, both in the presence or absence of H_2_O_2_ treatment (Fig. 6A). Likewise, induction by H_2_O_2_ of exon-1b containing transcripts of MDM2 was also blocked (Fig. 6A). When ncRNAs were analyzed, it was found that p53 knock-down significantly inhibited the induction of predicted p53 target lncRNAs MALAT, NEAT and PVT1 upon H_2_O_2_ treatment (Fig. 6B). miR-192-5p has been described to be a p53 transcriptional target in a variety of cancer cells [30-32]. However, miR-192-5p precursor transcripts were undetectable by small RNA-sequencing in HUVEC, escaping our analysis. Thus, we measured by qPCR whether the levels of mature miR-192-5p were affected by p53 silencing. Fig. 6B shows that, indeed, p53 knock-down prevented miR-192-5p induction in H_2_O_2_-treated HUVEC.

### Noncoding RNA modulation in senescent endothelial cells

**Figure 7:**
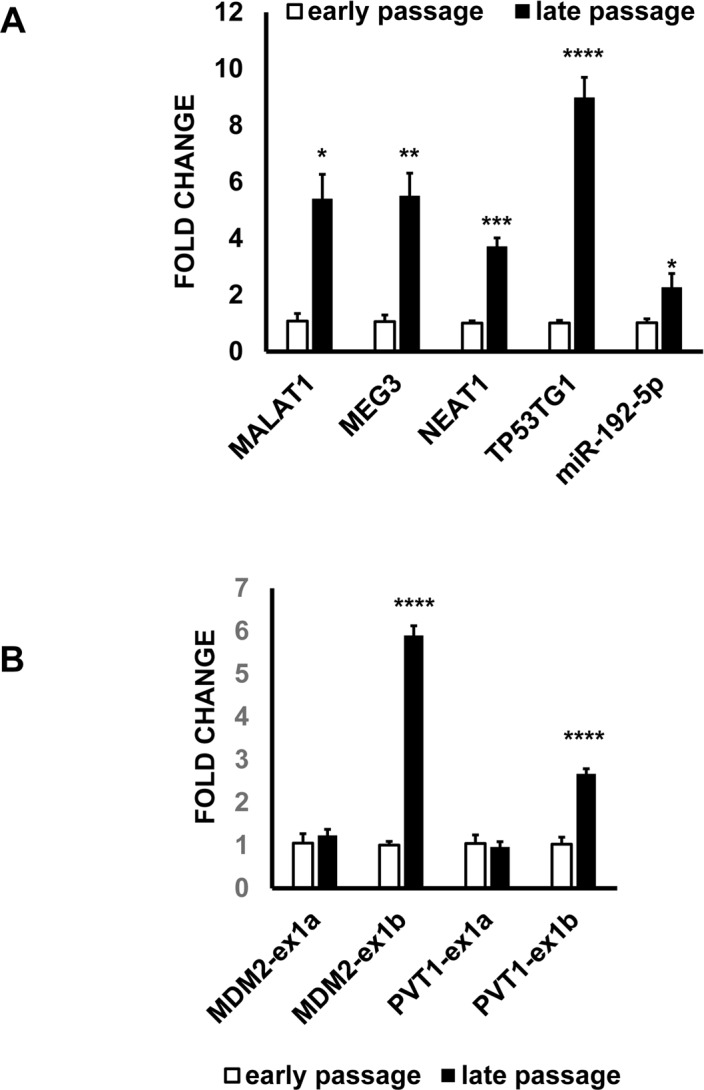
ncRNA alterations identified in H_2_O_2_-treated ECs are also present in senescent HUVEC Total RNA was extracted from early and late passage HUVEC and the indicated RNAs were tested by qPCR. The bar graphs represent average values ±SEM (early passage n= 3, late passage n= 7; ^*^p<0.05, ^**^p<0.01, ^***^p<0.001, ^****^p<0.0001). (**A**) ncRNAs. (**B**) Differential exon usage. Exon 1b isoforms of MDM2 and PVT1 are p53-regulated.

Next we wanted to assess whether the ncRNA alterations identified in H_2_O_2_-treated HUVEC were present also in other experimental systems characterized by altered redox balance. Specifically, replicative senescence is closely interlinked with increased oxidative stress, and it is characterized by a strong activation of the p53 stress-response pathway [6-8]. As expected [11, 35], late passage HUVEC displayed senescence markers, such as increased CDKN1A and GADD45A mRNA levels, decreased RAD51 mRNA and were mostly positive for senescence-associated β-galactosidase activity (Fig. S10). When a subset of H_2_O_2_-modulated lncRNAs and miRNA was assayed, they all displayed increased levels in late passage HUVEC (Fig. 7A). Moreover, both MDM2 and PVT1 exhibited a pattern of differential exon usage similar to that observed in response to H_2_O_2_ (Fig. 7B). Indeed, while exon 1a isoforms were not modulated, exon 1b isoforms of MDM2 and PVT1 were increased in senescent HUVEC.

### miR-192-5p increase in ischemic muscles

**Figure 8:**
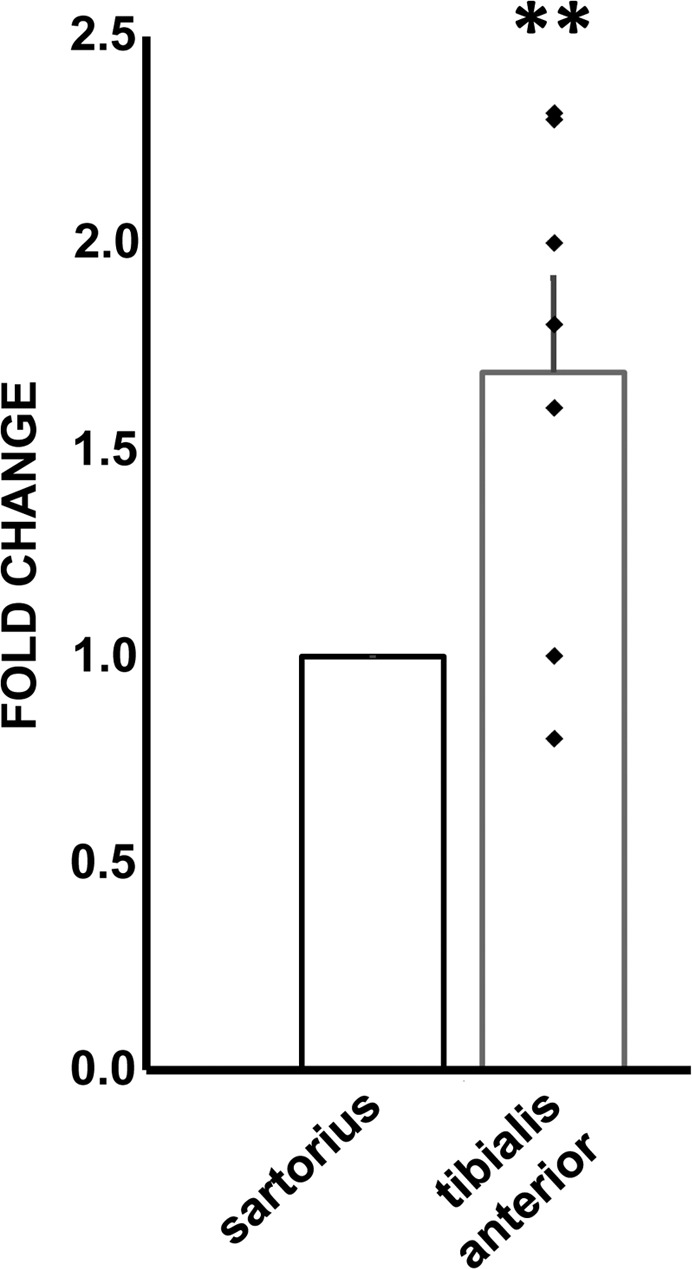
Increased miR-192-5p levels in ischemic muscles Skeletal muscle samples were harvested from the ischemic limb of patients undergoing above the knee amputations for CLI. Tibialis anterior and sartorious muscle samples were harvested and total RNA was extracted. The bar graph shows average ±SEM miR-192-5p levels measured by qPCR. Values of individual patients are indicated as black squares. For each individual, ischemic sartorius muscle values are referred to non-ischemic sartorius values (n= 7; ^**^p< 0.01).

Critical limb ischemia (CLI) is associated to increased oxidative stress levels in the ischemic muscles [9-11]. Thus, we investigated whether the ncRNA alterations identified in H_2_O_2_-treated HUVEC were present also in this system. Ischemic muscles are characterized by necrosis and inflammation, often precluding the possibility to isolate high quality RNAs. Thus, our analysis was limited to miRNAs, that are more resistant to degradation [11, 36], and in particular to miR-192-5p, the most significantly modulated miRNA in H_2_O_2_ treated ECs. Skeletal muscle samples were harvested from the ischemic limb of patients undergoing amputations for CLI. Specifically, ischemic tibialis anterior muscles were compared to non-ischemic sartorious muscle samples harvested at the amputation site of the same patient. Figure 8 shows that miR-192-5p levels were significantly increased in ischemic muscles.

miR-200c-3p was also tested, but was not detectable.

### Altered levels of H_2_O_2_-response RNAs in the peripheral blood of long living individuals

**Figure 9:**
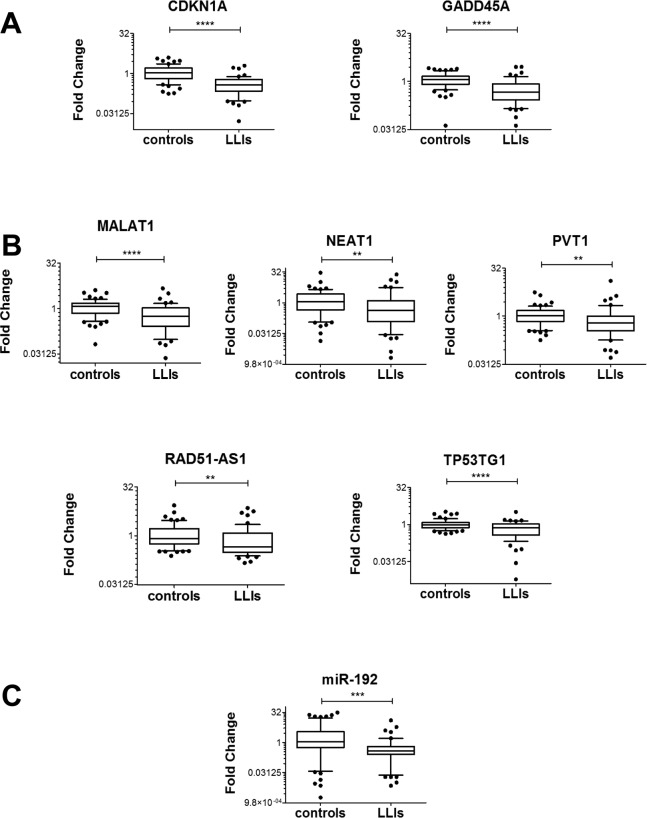
Association between H_2_O_2_-responsive RNA levels and life-span Box plots of the indicated RNAs in LLIs (n= 53) versus controls (n= 65); (^*^p≤0.05, ^**^p≤0.01, ^***^p<0.001, ^****^p<0.0001 after adjustment for sex distribution). (**A**) Coding RNAs. (**B**) LncRNAs. (**C**) miRNAs.

Oxidative stress response pathway has been linked to aging [12, 13] and the fine regulation of the stress response is crucial for healthy aging [37]. Thus, we hypothesized that long-living individuals (LLIs) might have moderate oxidative stress levels and, as a consequence, low levels of stress-response genes. To test this hypothesis, we measured the levels of coding and noncoding H_2_O_2_-response RNAs in the peripheral blood mononuclear cells (PBMCs) of LLIs [38, 39]. Specifically, 53 LLIs (98.2±4.1 years old) were compared to 65 controls (49.2±14.2 years old). Results show a global reduction of H_2_O_2_-response RNAs in LLIs (Fig. 9), with the exception of MDM2 isoforms, MEG3 and miR-200c-3p (Fig. S11).

**Figure 10:**
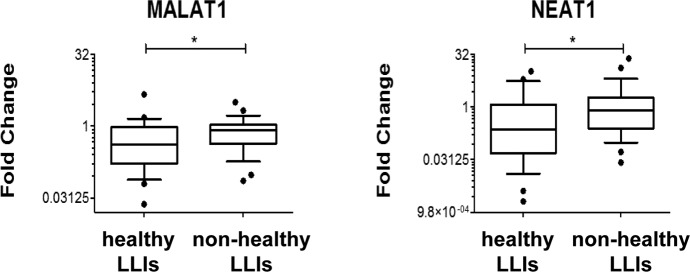
Association of MALAT1 and NEAT1 levels to healthy life-span Box plots of the indicated RNAs in LLIs subdivided based on the presence/absence of age-associated diseases (healthy n= 25; non-healthy n= 28; ^*^p<0.05 after adjustment for sex distribution).

Among the LLIs, 28 were frail (i.e. with age-associated disease) and frailty appears to be associated with higher oxidative stress levels [40]. Thus, significantly modulated RNA levels were compared between healthy and frail LLIs. It was found that H_2_O_2_-induced lncRNAs MALAT1 and NEAT1 levels were significantly lower in healthy LLIs (Fig. 10).

## DISCUSSION

In the present study, the differential expression landscape of protein-coding and non-coding transcripts in H_2_O_2_ treated ECs was outlined by next-generation sequencing.

H_2_O_2_ concentration used in this study induced a strong cytostatic effect and very low EC death *in vitro,* in keeping with previous studies [18, 19, 41-48]. Therefore, the ensuing redox imbalance is likely close or below to levels expected to occur in many pathophysiological conditions, such as reperfusion injury, when oxidative stress can induce not only growth arrest but also extensive cell death [49-51]. It is worth noting that other ECs might be more sensitive to H_2_O_2_. For instance, Park reported that H_2_O_2_ inhibited the growth of Calf Pulmonary Artery Endothelial Cells (CPAECs) and HUVECs at 24 h with IC50 of approximately 20 and 300 μM, respectively [52].

After filtering of rRNA-depleted RNA-sequencing datasets, we analyzed about 120 million of paired-end reads per sample, allowing the measurement of low expressed mRNAs and lncRNAs. Validation performed by qPCR on independent samples confirmed the accuracy of our analysis for all RNA species.

A recent study reported the transcriptome profiling of 3D co-cultured cardiomyocytes and human ECs under oxidative stress [53]. In the conditions adopted by the authors, a very limited number of genes was modulated in ECs, possibly due to the stabilizing action of the co-culture with cardiomyocytes.

Our analysis was also extended to miRNAs, facilitating the identification of miRNA/mRNAs interactions. Of note, results were not completely overlapping with a previous study performed by our group in H_2_O_2_-treated HUVEC, most likely due to differences in cell culture conditions and in profiling technologies, i.e. qPCR-arrays [19] *vs* next generation sequencing (this study). Indeed, miR-200c-3p and miR-1 found to be significantly modulated by qPCR-arrays [19], were not included in the small RNA-sequencing libraries, possibly also because very little expressed in ECs. On the other side, in the qPCR-array study [19] miR-192-5p was induced by H_2_O_2_ treatment, but not further pursued, miR-16-5p was not modulated, while probes for miR769-5p were not present in the array. Single miRNA qPCR assays for miR-200c-3p and miR16-5p fully reconciled the two investigations.

Data analysis showed a pivotal role of p53 [5] in EC response to oxidative stress and this pervasive p53 action was not limited to coding transcripts, but was extended to lncRNAs and miRNAs. Accordingly, many validated p53 target mRNAs were modulated and enriched biological processes and pathways included not only p53 signaling, but also many other related categories, such as cell cycle, DNA damage/stress response and apoptosis.

LncRNAs seem to be part of the p53-pathway as well. Indeed, among the H_2_O_2_-modulated lncRNAs in HUVEC, MEG3 has been found to be an activator of p53 in other systems [54, 55] and p53 induces formation of NEAT1-containing paraspeckles, that modulate replication stress response and chemosensitivity [56]. Moreover, MALAT1-depleted fibroblasts are sensitive to p53 levels, indicating that p53 is an effector of MALAT1 pro-proliferative activity [57].

Potential promoter analysis and survey of public p53 ChIP-seq datasets, indicated 29 lncRNAs modulated by H_2_O_2_ in ECs as p53-target candidates. Accordingly, when a subset of them was assayed in p53-silenced HUVEC, their induction by H_2_O_2_ was blunted. These observations are also in keeping with data in other experimental systems: indeed, PVT1 [26], NEAT1 [58] and TP53TG1 [59] have been found to be p53 transcriptional targets in a variety of cancer cells. Another lncRNAs activated by HUVEC treatment with H_2_O_2_ in a p53-dependent manner was MALAT1. However, the role played by p53 might be context dependent. Indeed, in mouse erythroid myeloid lymphoid cells undergoing differentiation, p53 binds to Malat1 promoter repressing its transcription [60].

It is worth noting that *NEAT1* and *MALAT1* loci are adjacent to one another in the genome. Independent p53-binding sites were present upstream their transcription start sites, but it is tempting to speculate that they might be functionally related.

Another important noncoding player in p53 pathway seems to be miR-192-5p. Indeed, in keeping with previous observations in cancer cells [30-32], miR-192-5p activation was p53-dependent; its expression, in turn, increased the levels of p53-targets, leading to EC growth arrest and death. Accordingly, evidence of a p53-miR-192-5p positive feed-back loop has been found also in multiple myeloma as well as in colon, breast, lung and ovary cancer cell lines [30-33, 61]. Finally, a similar feed –forward mechanism has been identified by our group for p53 and miR-200-3p as well [18, 19].

Bioinformatic analysis of the interactions between the identified miRNAs and their modulated predicted targets, indicated miR-192-5p as a potential hub of the EC response to H_2_O_2_. Interestingly, gene ontology analysis of miR-192-5p targets identified p53-related categories, such as cell cycle and DNA damage/stress response.

Library sequencing depth and the low expression levels of many transcripts precluded a high-throughput analysis of alternative exon usage events. Nevertheless we were able to show p53-dependent use of starting exons for both MDM2 [23] and PVT1 [26]. Exon 1b-containing isoforms of MDM2 mRNA are efficiently translated and their p53-driven transcription prompts p53/MDM2 negative feed-back loop [23, 24]. The functional consequences of PVT1 alternative 5′ exons usage are not clear to date, but it it is possible to speculate that it might change the transcript secondary structure.

We also showed that the lncRNA and miRNA alterations identified in H_2_O_2_-treated HUVEC were present also in other experimental systems where redox balance alteration plays a significant functional role. In particular, replicative senescence is closely interlinked with increased oxidative stress, shares many molecular mechanisms with H_2_O_2_-induced senescence and it is characterized by a strong activation of the p53 stress-response pathway [6-8, 62]. Indeed, all of the lncRNAs, miRNAs and alternative exon usage events identified in H_2_O_2_-treated cells that we tested were observed also in senescent EC.

The molecular mechanisms identified in ECs cultured *in vitro* might be also important in physiological and pathological conditions in humans. CLI is associated to increased oxidative stress levels in the ischemic muscles and redox imbalance plays a causal role in ischemia-induced tissue damage [9-11, 63]. We found that miR-192-5p levels were increased in ischemic muscles of CLI affected patients. The anti-proliferative and pro-apoptotic activity of miR-192-5p relates with the poor regenerative and cell death milieu observed in the muscles of CLI patients [10]. Unfortunately, low patient numerosity precluded miR-192-5p correlation with clinically relevant parameters.

Another relevant condition is aging. The deterioration with age of the stress response mechanisms increases the risk of many age-related diseases. In contrast, LLIs display preserved stress response mechanisms, likely derived from a combination of a healthy lifestyle and a favorable genetic background. The integration of these factors could allow LLI to maintain moderate oxidative stress levels that exert beneficial signaling and modulatory effects on cellular metabolism [37].

Accordingly, we found that out of 12 coding- and noncoding-RNAs tested in PBMCs, most displayed decreased levels in LLIs compared to control individuals. While several interpretations are possible, the simplest explanation is that the moderate oxidative stress levels characterizing LLIs are associated to lower levels of RNA species involved in the cell response to redox imbalance. In this respect, the levels of MALAT1 and NEAT1 seem to be particularly informative, since they displayed decreased levels in healthy compared to frail LLIs, emerging as potential biomarkers of healthy aging.

While the functional interaction between MALAT1 and NEAT1 is still largely unknown, one might speculate that they might play separate, but complementary roles in cell response to stress. These abundant nuclear enriched transcripts share some features [15, 64] and are both hypoxia-induced [65, 66]. MALAT1 localizes to nuclear speckles, which are nuclear bodies enriched for serine and arginine-rich splicing factors. NEAT1 is required for the formation of a different type of nuclear bodies, named paraspeckles for their close proximity to speckles. Of note, paraspeckles have been implicated in sequestering RNAs that respond to cellular stress [15, 64]. Thus, low MALAT1 and NEAT1 in healthy LLIs likely correlates with reduced cellular stress.

NEAT1 and MALAT1 co-enrich a number of *trans* genomic binding sites and protein factors [67]. However, MALAT1 primarily localizes within gene bodies and near the transcriptional start-sites, while NEAT1 localizes preferentially to transcriptional start- and termination-sites, suggesting independent but complementary roles for these RNAs in regulating nuclear bodies organization [67].

In conclusion, a RNA-sequencing approach was used to identify protein-coding and non-coding RNAs modulated in human ECs exposed to H_2_O_2_. We showed that these findings were not limited to this specific setting but could be extended to other conditions, such as proliferative senescence, CLI and healthy aging. Thus, the present study may contribute further insight into the biological functions and molecular mechanisms of lncRNAs and miRNAs in physiological and pathological conditions where redox imbalance plays a causal role.

## MATERIALS AND METHODS

### Cell cultures and transfections

HUVEC (Clonetics for profiling experiments or Thermo Fisher for all the other experiments) passage ≤4 were cultured in EGM-2 (Cambrex) at 37°C in a humidified atmosphere of 5% CO_2_- 95% air. N-acetyl-L-cysteine (NAC, Sigma) and 1,3-bis(2 chloroethyl)-1-nitrosourea (BCNU, Sigma) were dissolved in water.

HUVEC were transfected with 60 picomoles of a mix of TP53HSS110905/TP53HSS186391/TP53HSS186390 Stealth p53-RNAi, with 50 picomoles of hsa-miR-192-5p mirVana miRNA mimic (Life Technologies) or with negative control #1 (Life Technologies) using siRNA transfection reagent (Santa Cruz Biotechnology) in 40% confluent HUVECs, according to the manufacturer's manual. When relevant, cells were exposed to 200 μM H_2_O_2_ 24 hrs after transfection.

In replicative senescence experiments, early and late passage HUVEC underwent 8 and 18 population doublings, respectively. Population doublings were calculated as described previously [68]; briefly, the number of population doublings that occurred between passages was calculated according to the following equation: population doublings = log_2_(Ch/Cs), where Ch is the number of viable cells at harvest and Cs is the number of cells seeded.

### Flow cytometry

HUVEC were incubated for 30 min with 20 μM BrdU (Sigma) and then fixed with 70% ethanol. Cell cycle analysis was performed by combined anti-BrdU (Becton Dickinson) and propidium iodide (PI) staining using a Becton Dickinson flow cytometer. Cell Quest software was used to determine the percentage of BrdU-positive cells.

### Library preparation and RNA-sequencing

Total RNA was extracted as previously described [69] using TRIzol reagent according to the manufacturer's instructions (Thermo Fisher Scientific Inc.). The purity and integrity of the obtained RNAs was measured by Nanodrop (Thermo Fisher Scientific Inc.) and Bioanalyzer (Agilent Technologies). For mRNA and lncRNA analysis, rRNA depletion and library preparation were performed following manufacturer instruction for the Nugen OVATION RNA-Seq System V2 and Nugen OVATION Ultralow Library Systems (rRNA-depleted RNA-sequencing). For miRNA analysis, extraction and library preparation were performed following manufacturer instruction for the TruSeq Small RNA Library Preparation Kit. An Illumina HiSeq 2500 was used to perform a paired-end sequencing with 100 bp long reads (small RNA-sequencing). The average estimated insert size for mRNA and lncRNA was 150 bp. Small RNA libraries were sequenced twice.

### RNA-sequencing data analysis

FastQC (http://www.bioinformatics.babraham.ac.uk/ projects/fastqc, version 0.11.3) was used for quality control for both long and short RNA datasets. For mRNA and lncRNA analysis, datasets were aligned on human hg19 reference genome using STAR [70] aligner (version 2.5.3a). Gene level differential expression analysis was done counting on hg19 annotation using FeatureCounts (https://www.r-project.org/) [71] in the Rsubread package and using the counts for a standard limma-voom [72] analysis. LncRNAs were identified using the biotypes indicated by ENSEMBL. Genes, both coding and noncoding, displaying ≥1 cpm in at least 3 samples were considered as expressed.

For miRNA analysis, reads were trimmed using Trimmomatic [73] software set for trimming adapters and regions with low quality scores. Only one mate for each pair and only reads longer than 15bp were retained. Datasets were aligned on human hg19 reference genome using BWA [74] aligner (version 0.7.12-r1039) and miRNAs differential expression was calculated using mirBase v20 database [75] and a standard limma approach. Linear model was set to take into account both the batch effect within the three available HUVEC batches and between two sequencing rounds. miRNAs displaying ≥ 10 reads every 10M reads in at least 3 samples in both sequencing rounds were considered as expressed.

For all RNA species, events were defined as significant using a cutoff of adjusted p value <0.05.

### Data availability

Sequencing libraries are available on Gene Expression Omnibus [76] database under the accession number GSE104666. The superseries links to both the long and short RNA datasets. Available data comprise raw FASTQ and processed counts.

### Gene expression bioinformatic analysis

Enrichment in GO terms from the Biological Process tree and KEGG pathways was calculated using ClueGO [21] app for Cytoscape [28], set to report terms with a corrected p value <0.05.

For MDM2 isoform-level differential expression analysis, DEXSeq [77] was used; results were confirmed using SwitchSeq (https://github.com/ mgonzalezporta/SwitchSeq) and transcript-level analysis by VastTools (http://vastdb.crg.eu) [78]. Events were defined as significant using a cutoff of p value <0.05. Further details on the regions involved and their coverage were manually gathered with the help of IGV browser [79].

For miRNA-target interactions, full list of known, validated miRNA-target interactions was downloaded from miRTarBase [27] website. The full database was intersected with the lists of significant miRNAs and mRNAs, retaining only miRNA-target couples showing opposite modulation in HUVEC cells under oxidative stress. Visual representation of the network of interaction was created in Cytoscape. Enrichment analysis was performed with ClueGO as described above on both the full list of modulated targets and on the targets of each single miRNA, separately.

### p53 ChIP-seq analysis

ChIP-Seq datasets for human U2OS osteosarcoma cells treated with nutilin were downloaded from GEO (GSE46641) [34].

Reads were aligned on human hg19 reference genome using Bowtie2 (version 2.2.8) and subsequently used for a standard narrow-peak detection analysis using MACS2 (version 2.1.1.20160309). Peaks were detected providing untreated as background and were reported only when having a FDR<0.001. Genomic sequence under the peak was extracted using custom R scripts. p53 consensus was downloaded from Jaspar [80] and matched to the genomic sequence under the peak using custom R scripts.

### Patient recruitment and classification

All studies were conducted in accordance with the ethical principles that have their origins in the Declaration of Helsinki.

For experiments with LLI subjects, 56 LLIs (older than 90 years of age) were enrolled by home visiting from community-dwelling people based in a rural area of southern Italy (Cilento) and anamnestic information was collected (Table S1) [38, 39]. Among LLIs, 28 presented systemic diseases (diabetes mellitus, hypertension, cardiovascular diseases, Alzheimer or senile dementia, respiratory diseases, rheumatoid arthritis) and were sub-classified as frail. Controls were 65 healthy donors recruited in the same geographic area of the LLIs, with an age range between 20 and 80 years old. All subjects were recruited in the same period of time and the collected blood samples were processed within 24h since collection. All subjects gave written informed consent to the study, which was approved by IRCCS MultiMedica Ethical Committee, CE/CE/42/2010/LDC, protocol N. “19 2010 Cardiovascolare”.

For experiments with CLI subjects, skeletal muscle biopsies were harvested from 7 patients, 3 males and 4 females, aged 74.4 ±4.6 years, affected by terminal CLI and/or gangrene of the leg and undergoing above the knee amputation. Samples were placed in RNAlater (Qiagen) and frozen or processed within 24 hrs. Ischemic tibialis anterior muscle samples were compared to sartorius samples harvested at the amputation site of the same patient. The study was approved by the IRCCS-San Raffaele ethical committee, protocol code miRNACLI, number 69/INT/2016.

### PBMC isolation

Peripheral blood (15mL) was collected through a forearm vein puncture and PBMCs were separated on Histopaque-1077 (Sigma-Aldrich) gradient at 1200xg.

### RNA extraction and qPCR

Total RNA from tissues and cultured cells was extracted using TRIzol (Thermo Fisher Scientific Inc.) as described previously (Greco 2017). For LLIs study, RNA was extracted using miRNeasy kit (Qiagen) following the manufacturer's instructions. Sizing, quantitation and quality of the extracted RNAs was checked by Nanodrop ND-1000 (Nanodrop, Thermo Fisher Scientific Inc.) and Bioanalyzer 2100 (Agilent Technologies).

For mRNA and lncRNA qPCR experiments, RNA was retro-transcribed using the SuperScript III Reverse Transcriptase kit (Thermo Fisher Scientific Inc.) according to the manufacturer's instructions. cDNAs were analyzed using the SYBR-GREEN qPCR method (Thermo Fisher Scientific Inc.) according to the manufacturer's instructions. Sequences of the primers are reported in Table S2. Data were normalized to 18s rRNA levels in LLI experiments and to average UBC and RPL23A levels for all other experiments. For miRNA qPCR experiments, miRNAs were measured using Taqman MicroRNA single assays (Applied Biosystems) as previously described [81]. Samples were normalized in H_2_O_2_-treatment experiments to miR-25 expression, identified as suitable by small RNA-sequencing data analysis. For all other experiments, miR-16 was used as normalizer.

For all RNA species, relative expression was calculated using the 2^−ΔΔCt^ method [82].

### Statistical analysis

GraphPad Prism v.4.03 software (GraphPad Software Inc.) and STATA 12 software (StataCorp. 2011. Stata Statistical Software: Release 12) were used for statistical analysis. Continuous variables were analyzed by Student's t-test, Mann-Whitney test or ANOVA, as opportune. Categorical variables were compared using the Fisher's exact test and the chi-squared test. For experiments with LLI patients, a linear regression model was used to detect the genes and miRNAs expression levels that resulted significantly associated with life-span and heath-span after adjustment by sex. Expression levels were analyzed as log_2_ values since their distribution was positively skewed.

All statistical tests were performed 2-sided and a p <0.05 was considered as statistically significant. Continuous variables were expressed as mean ± standard error of the mean (SEM).

## SUPPLEMENTARY MATERIAL















## Abbreviations

BrdU: bromodeoxyuridine
ChIP-seq: ChIP-sequencing
CLI: critical limb ischemia
EC: endothelial cell
FDR: False Discovery Rate
LLIs: long-living individuals
lncRNAs: long noncoding-RNAs
miRNAs: microRNAs
ncRNAs: noncoding-RNAs
PBMCs: peripheral blood mononuclear cells
SEM: standard error of the mean
