# Supplementary material for: Central role of the p53 pathway in the noncoding-RNA response to oxidative stress
Source: Aging (Albany NY). 2017 Dec 12;9(12):2559–86. doi: 10.18632/aging.101341 (PMC5764393; doi:10.18632/aging.101341)
Supplement: Supplementary file 1 [file aging-09-2559-s001.pdf]

SUPPLEMENTARY MATERIAL

Table S1. LLI study population (N=118).

Group	n	Age mean (SD)	Age range	Sex (M/F)
Controls	65	49.2 (14.2)	25-78	24/41
LLIs	53	98.2 (4.1)	91-107	18/35
Frail-LLIs	28	97.9 (3.7)	91-104	14/14
Healthy-LLIs	25	98.5 (4.5)	91-107	4/21

Non-Healthy-LLIs, frail long-living individuals affected with diabetes, hypertension, cardiovascular disease, Alzheimer's disease, senile dementia, respiratory diseases or rheumatoid arthritis; Healthy-LLIs, healthy-aged long-living individuals; LLIs, all long-living individuals.*LLIs vs controls p=0.7 Healthy vs Non-healthy LLIs p=0.01.

Table S2. qPCR primers.

**CDC25A**	**F**	AATGGGCTCCTCCGAGTCAA
	**R**	TTCTTTACTGTCCAATGGCCC
**CDKN1A**	**F**	TGAGCCGCGACTGTGATG
	**R**	GTCTCGGTGACAAAGTCGAAGTT
**FAS**	**F**	ATGCCCAAGTGACTGACATCAA
	**R**	CTGGAGGACAGGGCTTATGG
**GADD45A**	**F**	AATTCTCGGCTGGAGAGCAG
	**R**	CTTCGTACACCCCGACAGTG
**MDM2 exon1a**	**F**	CGAAAACCCCGGATGGTGAG
	**R**	CGAAGCTGGAATCTGTGAGGT
**MDM2 exon1b**	**F**	AGTGGCGATTGGAGGGTAGA
	**R**	GTCTCTTGTTCCGAAGCTGGA
**p53**	**F**	CCCCAGCCAAAGAAGAAAC
	**R**	AACATCTCGAAGCGCTCAC
**RAD51**	**F**	TCATACGCTAGCTGTCACCTG
	**R**	AGCAGCCGTTCTGGCCTAA
**C15orf5**	**F**	CTGGAGTGAGGAGACTGAGGA
	**R**	CTTTTAAACCCACACCGCTTGA
**DLEU2**	**F**	AGCCAAATGTCCCATTTAAAGTT
	**R**	ATTGCTGAGCTAAGTAGAGGTC
**GSN-AS1**	**F**	GCCCCTGTGTCATAGTCACC
	**R**	CACAGTTAAGTGAGGGCCCAG
**KIFC1**	**F**	GAGGCCTGACCAGATGGAAG
	**R**	GGCACTGTAGTGAGGGATGG
**LINC00702**	**F**	AGAAAGGAGAAGAAAGTAGCTTGT
	**R**	GAAGTGCCTGTAGGAGATTCCA
**MALAT1**	**F**	TGTGTGCCAATGTTTCGTTT
	**R**	AGGAGAAAGTGCCATGGTTG
**MEG3**	**F**	CTGGGTCGGCTGAAGAACTG
**NEAT1**	**F**	GGCACCAGCATTTTGTGGTT3
	**R**	ACAAGGCCTCAGAAATGGGG
**PVT1 exon1a**	**F**	CGGCACCTTCCAGTGGAT
	**R**	CCGTGTCTCCACAGGTCACA
**PVT1 exon1b**	**F**	TCCCGGAAGCTGCAGAAG
	**R**	CCAGGCCACGAGGTTTCTC
**PVT1 exon3_1 (fig S5)**	**F**	GGTGACCTTGGCACATACAG
	**R**	GTCCTGGCAGTAAAAGGGGAA
**PVT1 exon3_2 (fig S6)**	**F**	TGGCACATACAGCCATCATGA
	**R**	ACGTGCCAAGCAGCTCAAA

**RAD51-AS1**	**F**	GCGAGTTTACAGACTGCCCT
	**R**	GATGCATGCCGGGAGATGTA
**TMPO**	**F**	TGAAACTGAGGGAACAAGGAACA
	**R**	GCTCTGCCCTTTAGTGGTTCT
**TP53TG1**	**F**	ATTCCCAGTGAGCCGCTTTT
	**R**	GCTCTCAGAGTCCTTGGTGG

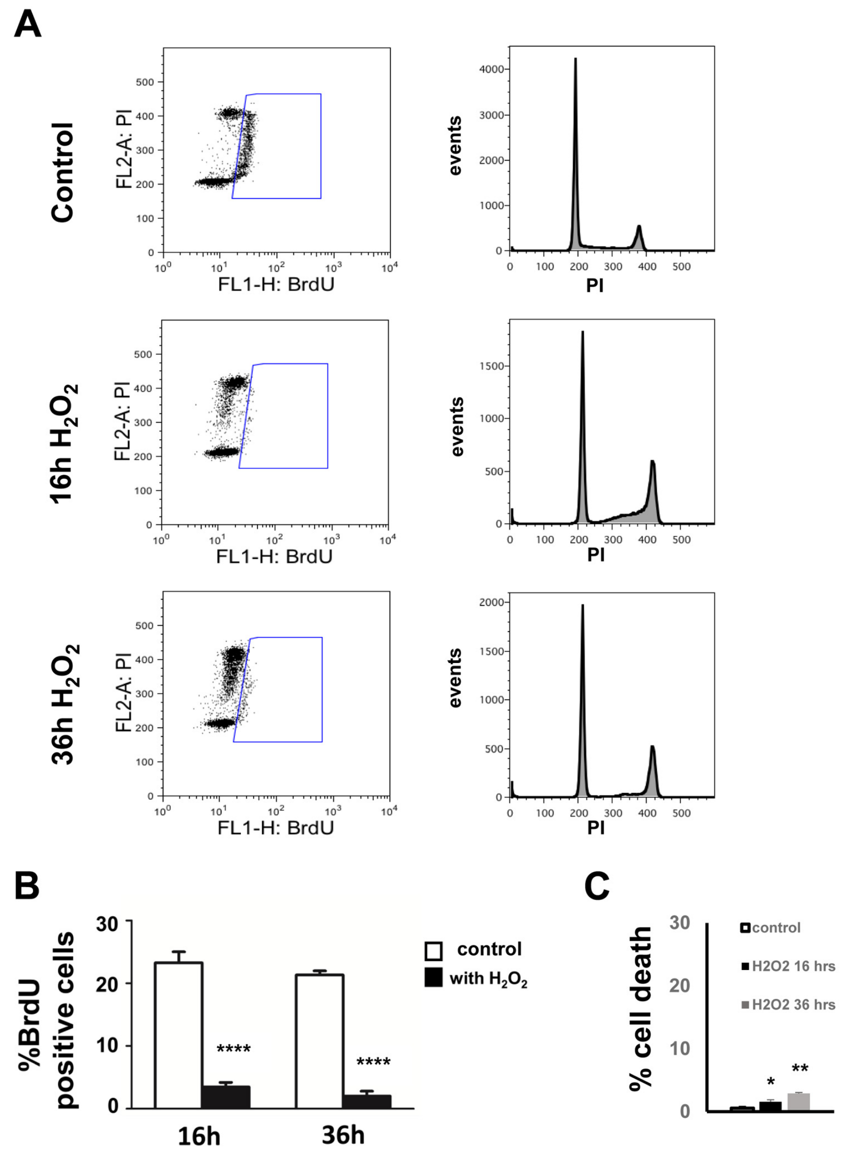

Figure S1. **Cell cycle analysis of H_2O_2–treated HUVEC used for RNA-sequencing profiling.** HUVEC were cultured with or without 200 μM H_2O_2 for 16 hrs and 36 hrs. Afterwards cells were pulse labelled with BrdU for 30 min before collecting, fixed and then stained with PI and α-BrdU antibody. (**A**) Representative images of BrdU incorporation (left panels) and PI staining (right panels), assessing DNA synthesis and DNA content, respectively, of HUVEC treated or not with H_2O_2 for 16 or 36 hrs. Pre-gated single cells were visualized in bivariate dot plots of BrdU vs PI to properly set the BrdU-positivity gates, thus taking into account the different autofluorescence of cells in the distinct phases of their cell cycle (left panels). Univariate histograms of DNA-bound PI are also showed (right panels). (**B**) Bar graph representing average values ±SEM of BrdU incorporation shows a decrease in DNA synthesis in H_2O_2-treated HUVEC (n= 3; ***P<0.001). C) Bar graph representing average percentage ±SEM of sub-G1 cells shows a significant but small increase of cell death upon H_2O_2 treatment (n= 3; *p<0.05, **p<0.01).

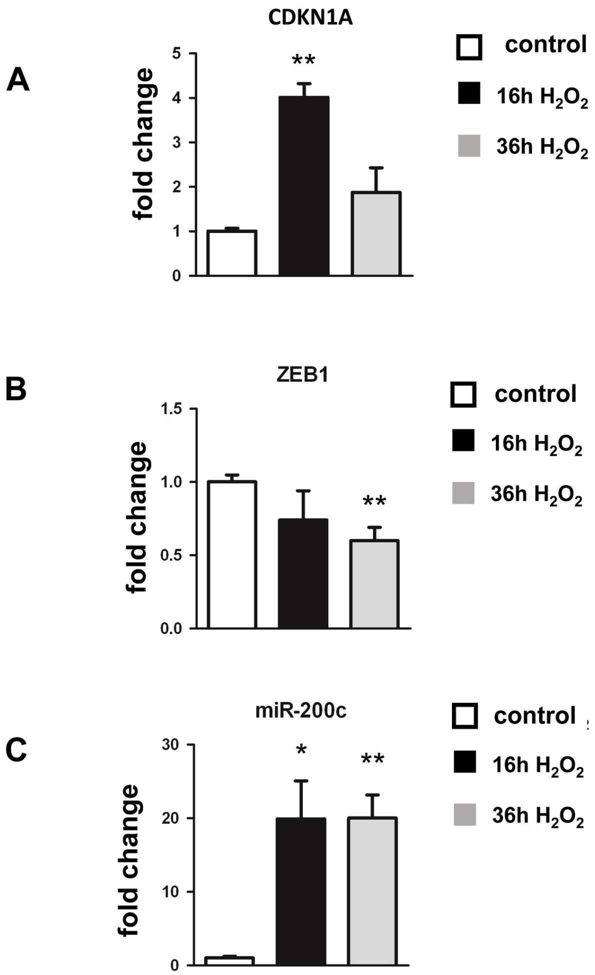

Figure S2. Positive controls in H_2O_2–treated HUVEC used for RNA-sequencing profiling. HUVEC were cultured with or without 200 μM H_2O_2 for 16 hrs and 36 hrs. Afterwards, total RNA was extracted and the indicated genes measured by qPCR. Average values are indicated ±SEM. A) The bar graph shows a significant increase of CDKN1A mRNA by 16 hrs of H_2O_2 treatment (n= 3; ***p<0.001). B) The bar graph shows a significant decrease of ZEB1 mRNA at 36 hrs of H_2O_2 treatment (n= 3; *p<0.05). C) The bar graph shows that miR-200c-3p was induced by H_2O_2 treatment (n= 3; *p<0.05; **p<0.01).

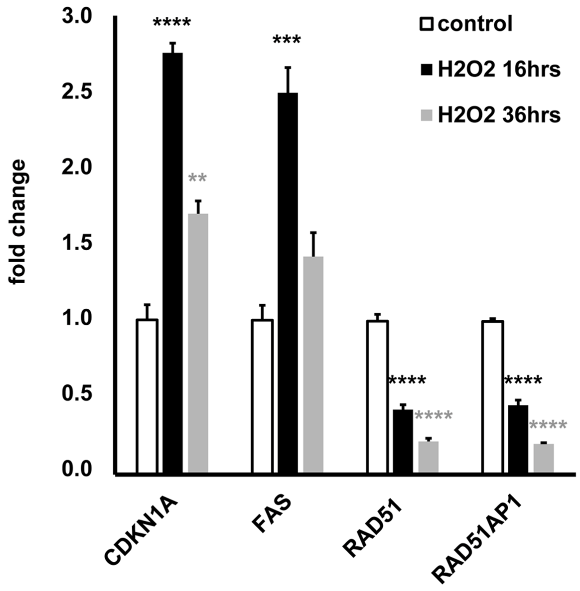

Figure S3. Validation of mRNAs differentially expressed upon HUVEC exposure to H_2O_2. HUVEC were exposed to H_2O_2 for 16 hrs and 36 hrs and the expression of the indicated mRNAs was measured by qPCR. The bar graph shows average ±SEM fold change values (n= 3; **p<0.01, ***p<0.001, ****p<0.0001).

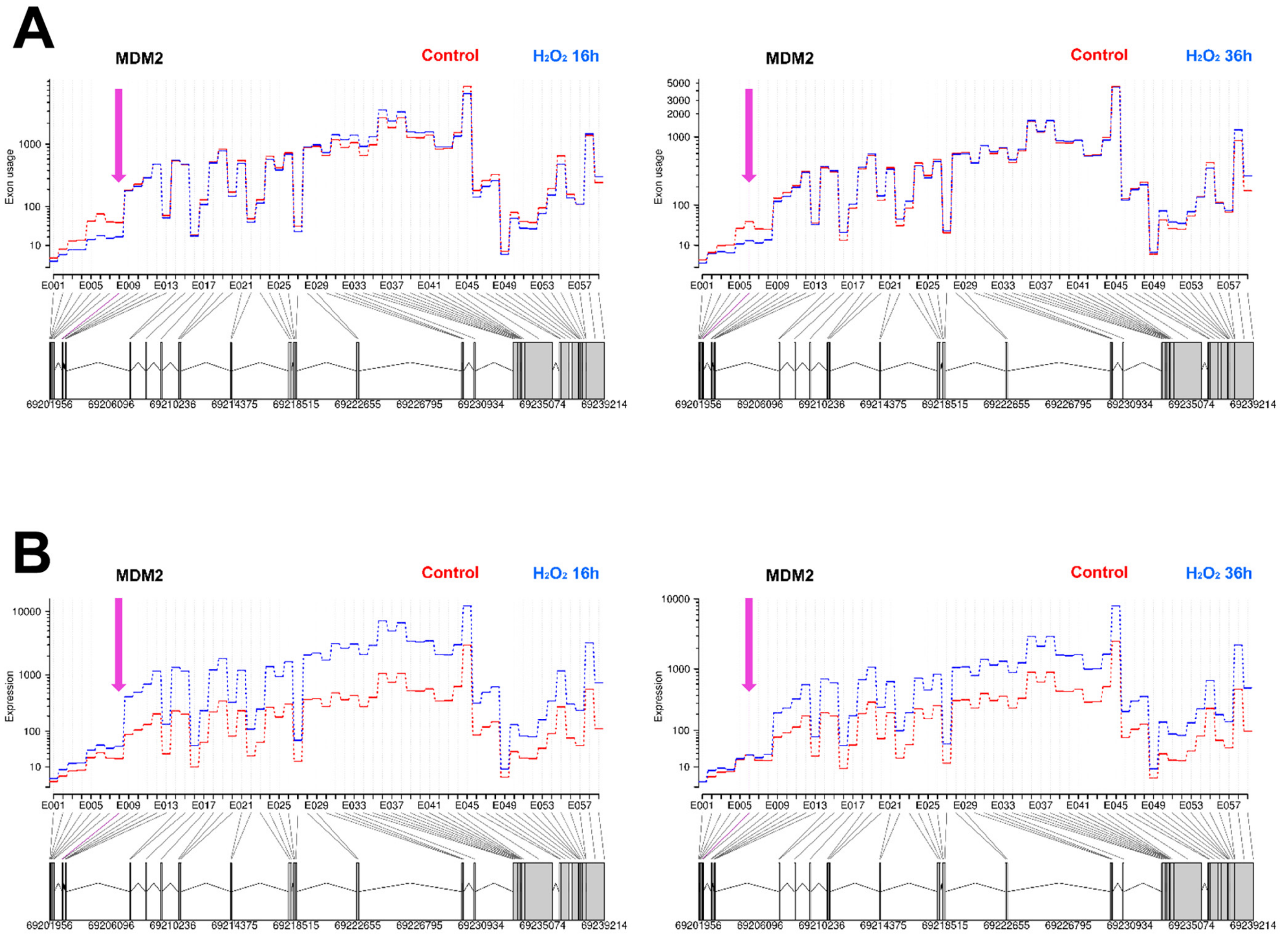

Figure S4. Alternative exon usage at the 5'UTR of MDM2. DEXSeq analysis of RNA-sequencing data reports a significant differential exon usage for the MDM2 gene at both 16 and 36 hrs of H_2O_2 HUVEC treatment. (**A**) Mean exon-level expression across replicates for both conditions was plotted against a schematic of all annotated exon fragments involved in a splicing event. The exon-level expression was in accord with the gene-level results that show a generalized increase of MDM2 gene in oxidative stress conditions. Expression upon H_2O_2 exposure was increased for all MDM2 exons but those at the 5' of the gene. (**B**) The same data are visualized normalizing expression values between all exon fragments of MDM2 to highlight differential exon usage at the 5' of the gene. Exon fragments displaying statistically significant differences are indicated by pink arrows (n= 3; p< 0.0001).

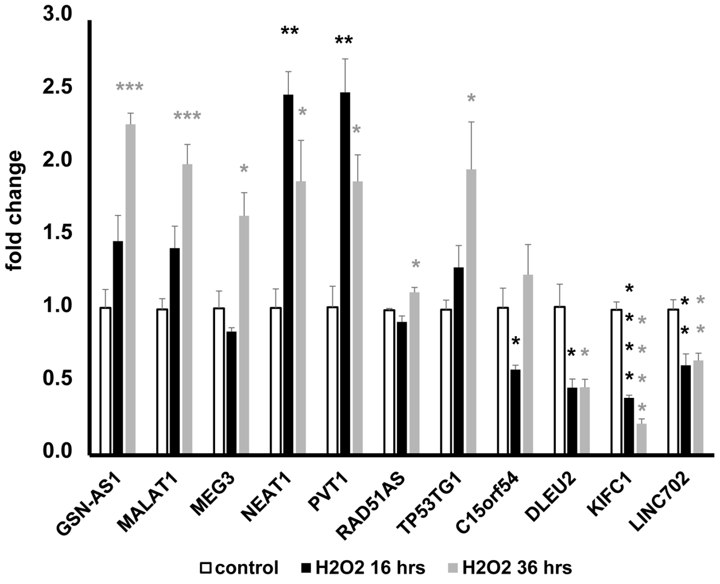

Figure S5. LncRNAs differentially expressed upon HUVEC exposure to H_2O_2. HUVEC were exposed to H_2O_2 for 16 hrs and 36 hrs and the expression of the indicated lncRNA was measured by qPCR. The bar graph shows average ±SEM fold change values expressed in a linear scale (n= 3; *p<0.05, **p<0.01, ***p<0.001, ****p<0.0001).

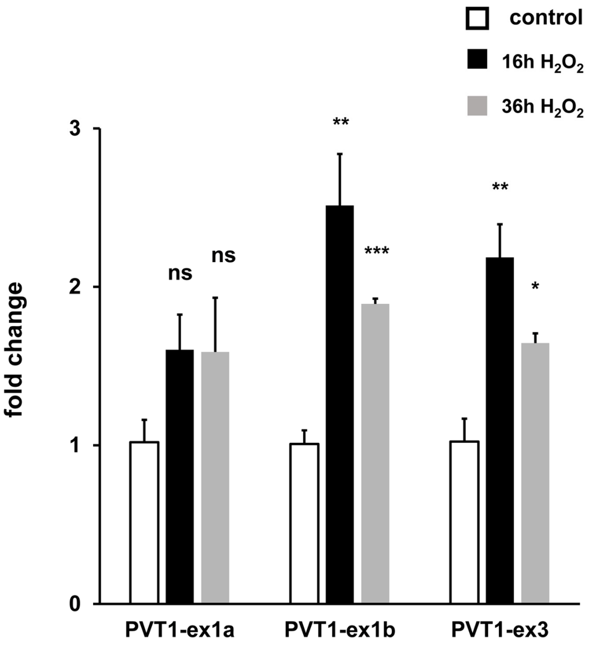

Figure S6. PVT1 alternative exon usage. HUVEC were exposed to H_2O_2 for 16 hrs and 36 hrs and the expression of the indicated PVT1 isoforms was measured by qPCR. In H_2O_2 treated HUVEC, exon 1b containing isoforms were induced. Modulation of exon 1a containing isoforms of PVT1 was not statistically significant. Primers detecting internal exon 3 were used as positive control. The bar graph shows average ±SEM fold change values (n= 3; *p<0.05, **p<0.01, **p<0.001).

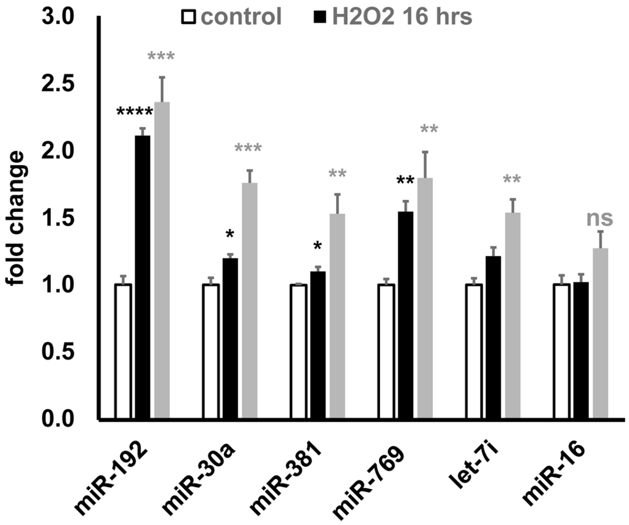

Figure S7. miRNAs differentially expressed upon HUVEC exposure to H_2O_2. HUVEC were exposed to H_2O_2 for 16 hrs and 36 hrs and miRNA expression was measured by qPCR. The bar graph shows average ±SEM fold change values expressed in a linear scale (n= 3; *p<0.05, **p<0.01, ***p<0.001, ****p<0.0001, ns= not significant).

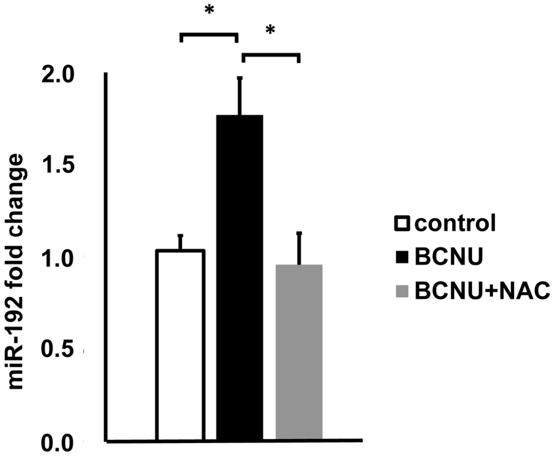

Figure S8. Redox imbalance induced by BCNU treatment induces miR-192-5p expression. HUVEC were either preincubated with 10 mM NAC or sham-treated for 30 min, followed by addition of 0.25 mM BCNU for 2 h. miR192-5p was measured by qPCR (n= 6; * p<0.05).

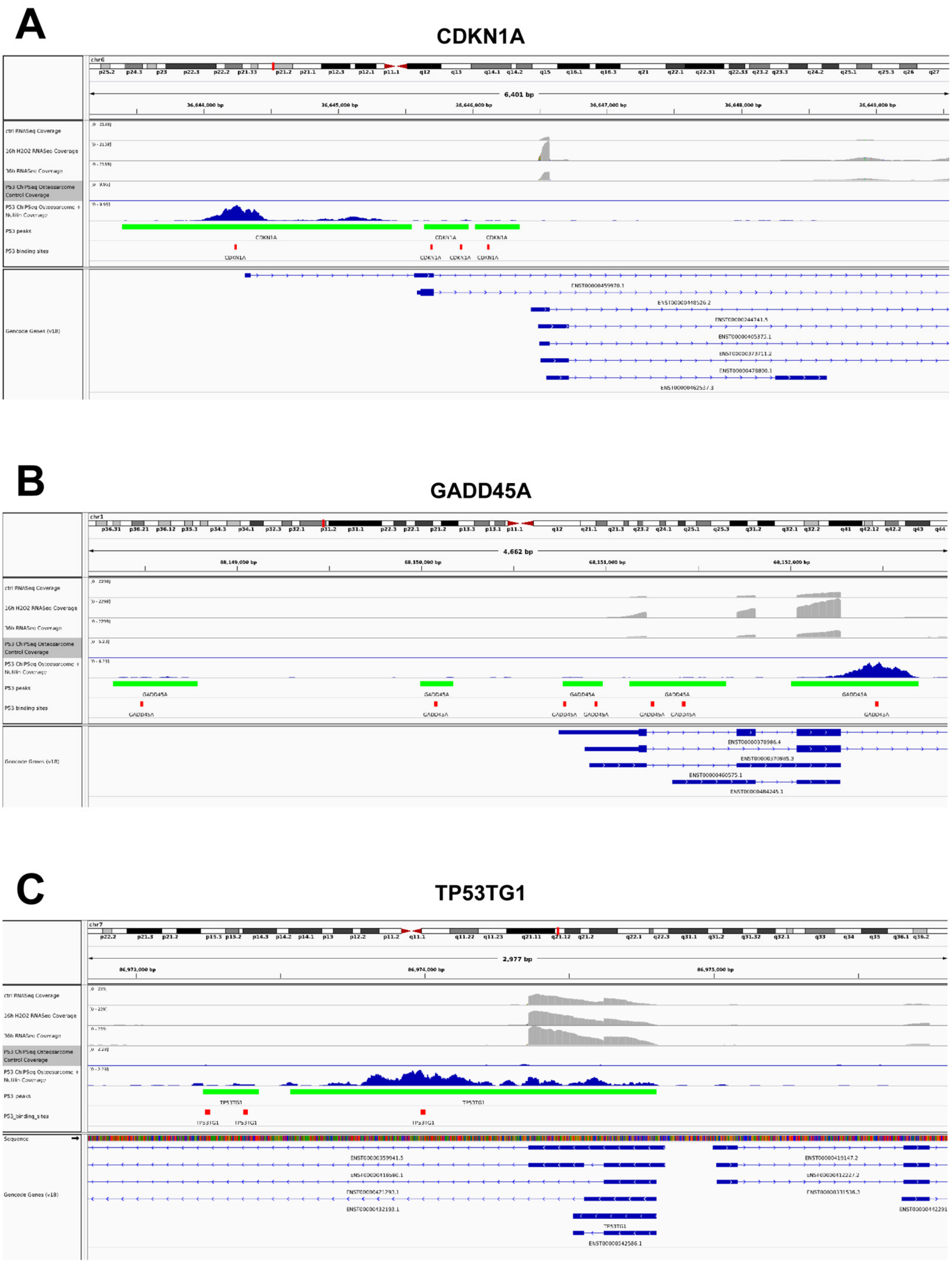
A
CDKN1A
chr6
6,401 bp
B
GADD45A
chr1
4,662 bp
C
TP53TG1
chr7
2,977 bp

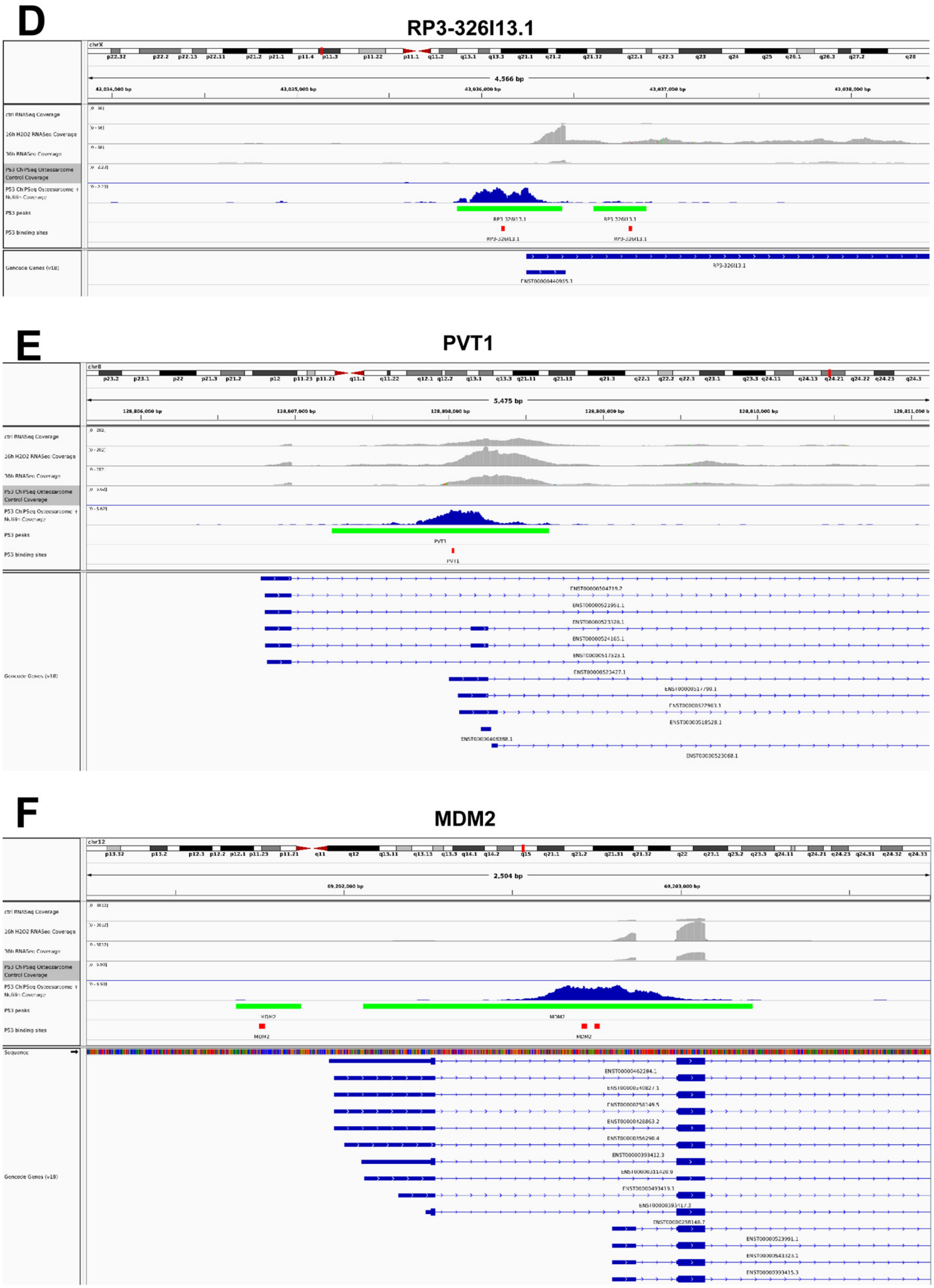

Figure S9. p53 occupancy in the regulatory regions of H_2O_2 modulated RNAs. Publicly available ChIP-seq data for p53 in osteosarcoma cells treated with nutilin, an activator of p53 (GSE46641) were analyzed. For each gene, the corresponding panel shows: genomic coordinates; reads in HUVEC treated with H_2O_2 for 0 hrs (ctrl), 16 hrs or 36 hrs (in gray); p53 ChIP-seq reads in control and nutilin treated osteosarcoma cells (in blue); significant (FDR<0.001) ChIP-seq peaks (green bars); consensus p53 binding sites (conservation ≥60%, in red); gencode annotated transcripts (in blue).

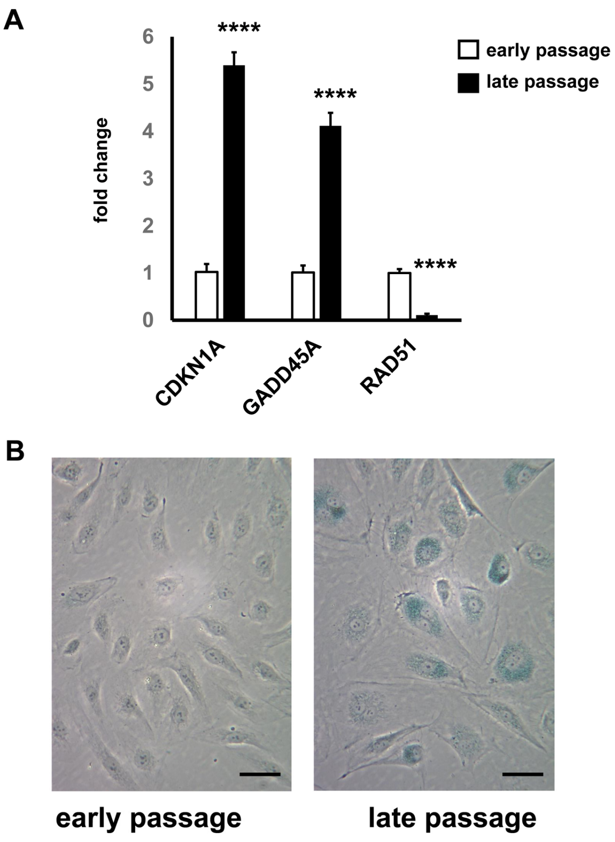

Figure S10. Senescence marker induction in late passage HUVEC. (**A**) Total RNA was extracted from early and late passage HUVEC and the indicated mRNAs were tested by qPCR. The bar graph shows average ±SEM fold change values (early passage n= 3, late passage n= 7; ****p<0.0001). (**B**) Representative pictures of early and late passage HUVEC stained for senescence-associated β-galactosidase activity (reference bar= 50μm).

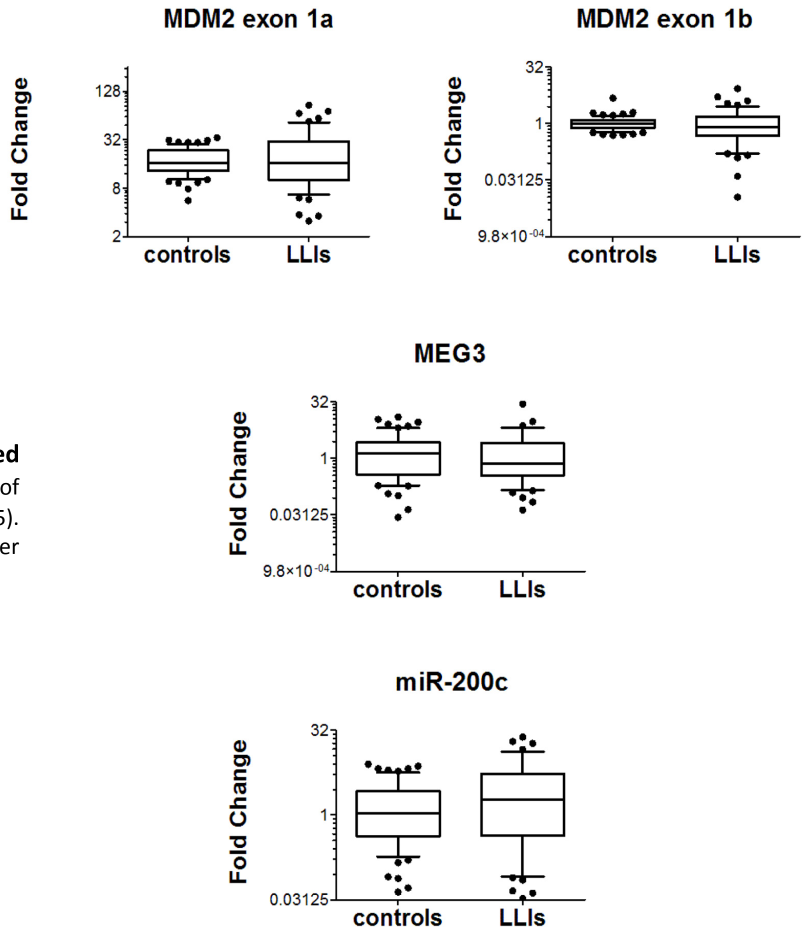

Figure S11. Lack of association between the indicated H_2O_2-responsive RNA levels and life-span. Box plots of the indicated RNAs in LLIs (n= 53) versus controls (n= 65). None of the differences reached statistical significance, after adjustment for sex distribution.
